# Extracorporeal Shockwave Therapy (ESWT) benefits in spastic children with Cerebral Palsy (CP)

**Published:** 2014

**Authors:** A Mirea, G Onose, L Padure, E Rosulescu

**Affiliations:** * ”Dr. Nicolae Robănescu” National Centre of Neuro-Psychomotor Clinical Rehabilitation, Bucharest, Romania; ** ”Carol Davila” University of Medicine and Pharmacy, Bucharest, Romania; *** “Bagdasar-Arseni” Clinical Emergency Hospital, Bucharest, Romania; **** Craiova University, Department of Kinesiotherapy and Sports Medicine, Craiova, Romania

**Keywords:** cerebral palsy, spasticity, shockwave therapy

## Abstract

**Introduction**. ESWT refers to the use of Shock Waves in medical practice. It was used as an important tool in spasticity management of children with CP. The aim of our study was to evaluate the effect of a 3 session of ESWT on spastic upper and lower limbs muscles in children with CP.

**Methods**. Sixty-three children (37 boys and 26 girls), mean age 99.57±53.74 months, were included in the study. We used focused ESWT, applied in 3 sessions during the admission of each child, on the mainly affected muscles, using the same parameters on all patients (energy – 0.15 mJ/mm2, shot dose - 500 shocks/ session, frequency - 10 Hz). All patients were assessed two times: once, in admission (before any physical or ESWT appliance) and second, at discharge (after receiving the entire prescribed treatment), following: Modified Ashworth Scale (MAS), Gross Motor Function Classification System (GMFCS), Gross Motor Function Measure 66 (GMFM-66) and also a Questionnaire on Pain caused by spasticity (QPS).

**Results**. We found a better and significant decrease of MAS level in the ESWT treated group, thus leading to a concomitant decrease of QPS score and also increase of GMFM-66 score.

**Conclusion**. ESWT, applied in 3 sessions, with 0.15 mJ/ mm2, using 500 shocks/ min and 10 Hz as frequency may decrease children spasticity level and pain caused by it and improve the gross motor function.

## Background

Cerebral palsy (CP) represents the motor impairment caused by the damage of a developing brain [**1**]. Children with CP are currently directed for physical therapy, but there is confusion and debate in the research and clinical literature about the efficiency of treatments, and about the relationship between the intensity of treatment and consequent benefits. The injury that occurs to the motor areas may lead to spasticity (around 80% of children), which is often accompanied by pain, being a major cause of disability, affecting daily activities and quality of life. Spasticity is a common finding in children with upper motor neuron syndrome in context of CP, being commonly defined as “a motor disorder characterized by velocity-dependent increase in the tonic stretch reflexes (“muscle tone”) with exaggerated tendon jerks resulting from hyper-excitability of the stretch reflex” [**2**]. The other (non-motor) CP related disorders, as impaired cognition, behavioral problems or hearing disorders may compromise communication, spasticity related pain being often underreported and thus undertreated [**3**].

Conventional treatment of spasticity may include passive stretching [**4**], serial plastering and splints [**5**], pharmacologic treatment [**6**], and botulinum toxin [**7**]. From a theoretical point of view, shock waves could be useful to treat spasticity in patients with upper motor neuron syndrome. In accordance with the effects on tendon diseases, shock waves could have a direct effect on muscle fibrosis and other non-reflex components of muscle hypertonia [**8**]. Furthermore, shock waves acting at the muscular level could modify the sensory inflow from the treated muscle to the spinal cord, thus reducing spinal cord excitability and mitigating spasticity [**9**].

**Fig. 1:**
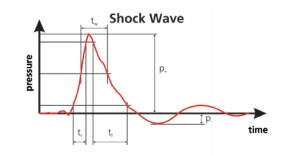
Pressure curve profile for SW [**10**]

Shock Waves (SW) are mechanical beats that need a material medium to propagate in order to convey. Extracorporeal shockwave therapy refers to the medical use of SW. Sound pressure waves, generated with a high-intensity level outside the human body underlie ESWT. When generated, it raises very quickly (just a few nanoseconds) to a high positive pressure peak (around 150 MPa), followed almost instantly by a negative one (around 5 MPa). The pulse (**Fig. 1**) lasts for around 0.3-0.5 microseconds [**10**]. The pulse energy needs to be focused in order to be applied where treatment is needed. In industry, there are three methods, applied to generate shockwave: electrohydraulic, electromagnetic and piezoelectric principles.

According to the International Society for Medical Shockwave Treatment (ISMST) [**11**] the following are the approved standard indications for ESWT: chronic tendinopathies (plantar fasciitis with or without heel spur, achilles tendon, radial epicondylopathy - tennis elbow, rotator cuff with or without calcification, patella tendon, greater trochanteric pain syndrome), impaired bone healing function (delayed bone healing, stress fractures, early stage of avascular bone necrosis - native X-ray without pathology, early stage osteochondritis dissecans post-skeletal maturity) and urology (lithotripsy – both extracorporeal and endocorporeal). ISMST also emphasizes the common empirically – tested clinical uses of ESWT [**11**] in tendinopathy (ulnar epicondylopathy, adductor syndrome, pes anserinus syndrome, peroneal tendon syndrome), muscular pathology (myofascial syndrome - fibromyalgia excluded, Injury without discontinuity), impaired wound healing, burn injuries and salivary stones.

ESWT’s intimate effects, from the genic-molecular transcriptional level to modulations within cell cycle’s functioning and respectively, to the nutritional- circulating/ reparative tissue one, especially in this decade, determined the appearance of some new ESWT indications, thus supplementary enlarging the spectrum of its clinical applications. One of these is the spasticity. The presumed action modalities of ESWT [**10**] may refer to a direct modulation of rheologic properties of the muscular tissue: tixotropy promoting (and thus braking the temporary functional links between the molecule of actins and myosin); retractile fibrosis preventing or even reducing within myo-elastic tissue - mainly through the vascular-metabolic link, associated with nutrition and of tissue clearance improvement and Ph, respectively.

Another assumptive action modality [**12**] may concern modulation of the activation threshold of the neural-muscle spindle: small muscle afferents capsaicin-sensitive fibers may influence the muscle spindle activity, and the peripheral rhythmic sensory signals - including of ESWT - control and inhibit the dorsal horn gate, thus down regulating the α motor neurons. Another and very tempting hypothesis [**13**] is Golgi tendon organ stimulation, with its inhibiting effect, by energetic stretch of it; but classically this is induced only after 6 seconds of muscle stretch.

Although the existing treatments proved their efficacy, we believe that shock wave therapy, as a non-invasive therapy may be an interesting alternative in the treatment of CP affected children spasticity. There are few studies that have assessed the efficacy of ESWT in CP; in a 2010 study the authors showed in a sample of children that FSWT produces a significant long-lasting reduction in spasticity in the plantar flexors [**14**]. Similar results were also found by a study that assessed the efficacy of RSWT in a sample of patients, mostly young adults, treating both the spastic muscles and their antagonists [**15**].

The aim of our study was to evaluate the effect of a 3 session of ESWT on spastic upper and lower limbs’ muscles in children with CP.

## Material and Methods

Sixty-three children (37 boys and 26 girls), mean age 99.57±53.74 months, were included in the study. All of the children had a diagnosis of spastic CP. Procedures and measurements were performed on inpatients of “Dr. Nicolae Robănescu” National Centre of Neuro-Psychomotor Clinical Rehabilitation, Bucharest, Romania.

The inclusion criteria for all children were the following: (a) informed consent signed by the parent; (b) age between 2-18 years; (c) diagnosed with spastic CP type; Ashworth score at least 1 and at most 3 in the targeted muscles.

The exclusion criteria were the following: (a) fixed contracture, defined as severe restriction of the range of joint movement on passive stretch or predominant forms of muscle hypertonia other than spasticity (i.e., dystonia) in the targeted muscles; (b) severe neurological associated disorders; (c) pure dyskinetic form of CP; (d) any muscle relaxant medication with peripheral action (i.e., intrathecal Baclofen) or botulinum toxin administrated within 2 weeks before the first evaluation and during the study; (e) any changes in the relaxant medication with central action within 2 weeks before the first evaluation and during the study.

Before the procedures, all parents were carefully informed about ESWT, its effects and side effects, and all signed an informed consent. The procedure was approved by the Ethics Committee of “Carol Davila” University of Medicine and Pharmacy.

Clinical examination was performed to assess the effect of the treatment. All children were assessed 2 times during the study: once, in admission (before any treatment) and second at discharge (after receiving the entire prescribed and accepted treatment).

The clinical assessment included the following: Modified Ashworth Scale (MAS), Gross Motor Function Classification System Expanded and Revised (GMFCS E&R) [**16**], Gross Motor Function Measure 66 (GMFM-66). The MAS grades spasticity according to six ordinal levels, from 0 ‘normal muscle tone’, to 4 ‘affected part(s) rigid in flexion or extension’ [**17**]. The Gross Motor Function Measure (GMFM) is a clinical tool designed to evaluate change in gross motor function in children with cerebral palsy, and has become one of the best evaluative measure of motor function designed for quantifying change in the gross motor abilities of children with cerebral palsy [**18**].

The pain associated with spasticity was challenging, especially because of children associated cognitive impairments, or children were too young to be interviewed. In this respect we used an Adapted Questionnaire on Pain caused by Spasticity (A-QPS), which includes questions and sad/ smiley faces (Wong-Baker FACES) to quantify pain intensity answers [**19**]. Thirteen pairs (children and parents) were enrolled for the cognitive interviews. The A-QPS included the following 5 modules (pain evaluation sections): (1) “Think of today and tell me if you feel any tension in the shoulder, arm, wrist, hip or foot. Please tell me how much you hurt. Below are some faces. Can you tell me which one shows best how much you hurt?”; (2) “Think of today, when you did not do anything special, just sit down/ watch TV/ trying to sleep. Did you feel any pain in shoulder, arm, wrist, hip or foot? How much did it hurt?”; (3) “Think of today, when you move, walk or play. Did it hurt your shoulder, arm, wrist, hip or foot? How much did it hurt?”; (4) “Think about today, when you were doing stretching exercises or physical therapy. Did you feel any pain in the shoulder, arm, wrist, hip or foot? How much did it hurt?”; (5) “Think of the most difficult movement that you can make with your shoulder, arm, wrist, hip or foot. How much does it hurt when you try to do this movement?”.

The classification of limb distribution for the hypertonic (primarily spastic) form of CP was accomplished in accordance with Surveillance of Cerebral Palsy in Europe registry classification [**20**].

The ESWT consisted in 3 ESWT sessions, BTL-5000 unit, using the following parameters: energy of 0.15mJ/mm2; total shots dose 500 shocks per each treated muscle; frequency 10 Hz; multi-focus hand piece with 15 mm diameter. The treatment was not painful and no anesthesia was needed.

A descriptive statistical analysis of the quantitative parameters of mean, SD and paired-sample t-test to compare the results before and after the treatment was carried out.

## Results

**Fig. 2:**
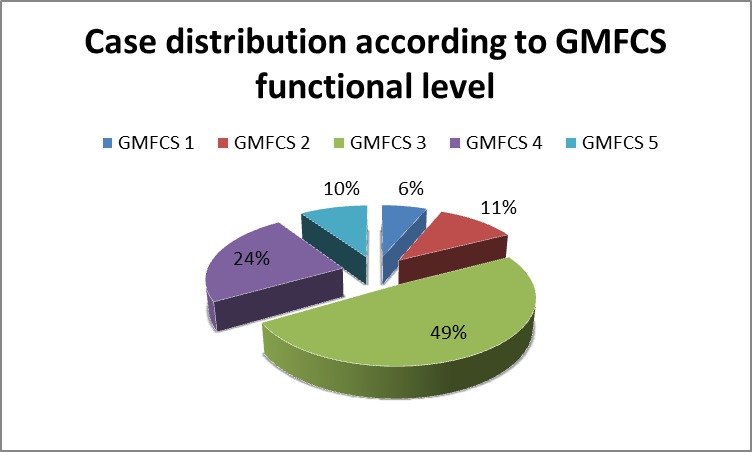
GMFCS case distribution

The GMFCS distribution of the cases: level I and II (being able to walk without and respectively with limitation) were found in 4 (6%) and 7 (11%) children. 31 (49%) children needed an assistive device (walker, wheelchair auctioned by themselves) to walk. 15 (24%) of the studied children and the rest, 6 (10%) were classified as level V, presenting severe motor dysfunctions (**Fig. 2**).

**Fig. 3:**
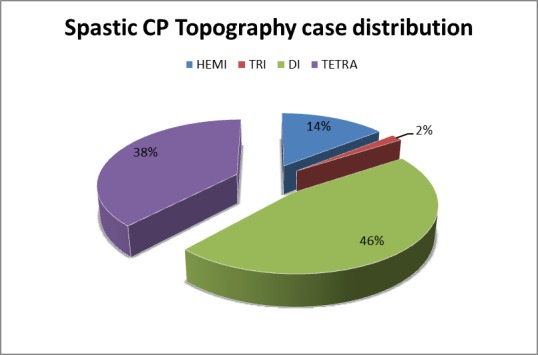
Spastic syndrome distribution

Spastic syndrome in the studied patients was distributed (**Fig. 3**) as it follows: quadriplegia was found in 24 (38%) children, hemiplegia in 9 (14%) cases, triplegia in 1 (2%) patients and most common was the diplegia, which was found in 29 (46%) children.

**Fig. 4:**
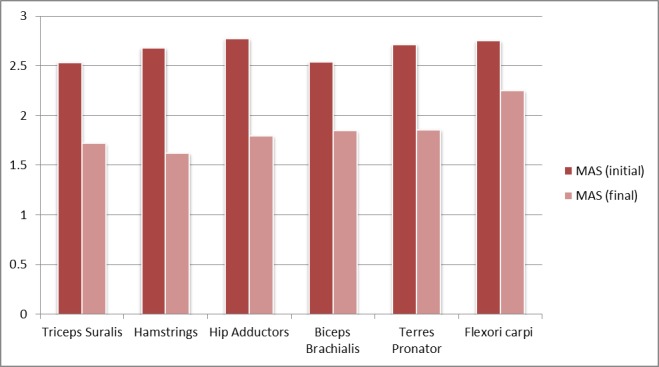
MAS evolution in the studied group, before and after ESWT

Spasticity assessment: in the studied group, ESWT proved to be efficient in the upper and lower limbs, reducing MAS with almost 1 degree (**Fig. 4**), thus contributing to the GMFM-66 improvement and consequently reducing the spasticity and related pain.

**Fig. 5:**
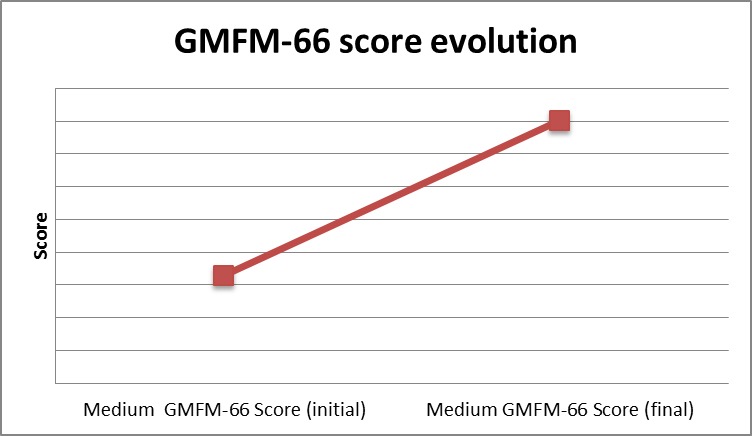
GMFM-66 score evolution

Gross motor evaluation: comparing the moments of assessment, we noticed a significant difference in GMFM-66 score evolution, which raised less more than 10 points in the studied group of children that benefited from ESWT (**Fig. 5**).

**Fig. 6:**
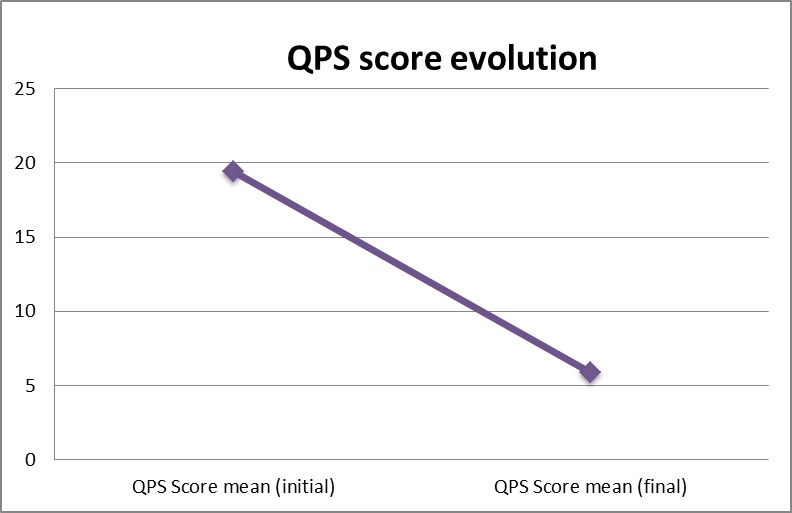
Comparison of QPS score evolution

 Pain assessment: the above-mentioned improvements led to a significant decrease (14 points in QPS evaluation) in spasticity related pain among the children from the studied group (**Fig. 6**).

## Discussion

This study presents the usefulness of ESWT in the treatment of spasticity in cerebral palsy children, as a noninvasive technique, painless and without important side effects. A recent randomized, placebo-controlled clinical trial assessed the benefits of RSWT in the treatment of spasticity in young adults with CP. In that study, the protocol consisted of three sessions of radial RSWT (with a 1-week interval between sessions) on different spastic muscles and on their antagonists in a small group of 15 patients, mean age 31 years (range 10–46) [**15**]. They also found a statistically significant decrease in MAS in the group treated with RSWT. Obtained clinical results were encouraging, thus the mechanism of shock wave therapy on spastic muscles is still unknown. A number of studies have investigated the effect of shock waves by inducing enzymatic and non-enzymatic nitric oxide synthesis [**21**]. We achieved a significant improvement after 3 sessions of ESWT in a homogenous group of CP children. We have to mention that only the spastic muscles and not their antagonists were treated, although there is evidence that the muscles with pathological changes could benefit from the application of shock wave therapy. We applied only 3 sessions and the results persisted for a 3-4 weeks. Basic research and further large randomized controlled studies are necessary to support the results of this clinical study.

## Conclusion

ESWT might efficiently improve spasticity in affected children and also their gross motor functionality. The spasticity related pain diminished in those children who received ESWT, thus improving these patients’ quality of life. Still, further ESWT studies are needed, on a larger population, in order to reveal, on one hand its mechanism of action and on the other to improve the methodology - on which mainly depends the therapeutic effects.
